# A randomized controlled trial of preemptive rituximab to prevent recurrent focal segmental glomerulosclerosis post-kidney transplant (PRI-VENT FSGS): protocol and study design

**DOI:** 10.3389/fneph.2023.1181076

**Published:** 2023-05-15

**Authors:** Michelle N. Rheault, Sandra Amaral, Margret Bock, Eileen Tsai Chambers, Blanche Chavers, Mireile El Ters, Rouba Garro, Rasheed Gbadegesin, Amit Govil, Lyndsay Harshman, Hatem Amer, David K. Hooper, Ajay K. Israni, Samy Riad, Junichiro Sageshima, Ron Shapiro, Michael Seifert, Jodi Smith, Randall Sung, Christie P. Thomas, Qi Wang, Priya S. Verghese

**Affiliations:** ^1^ Department of Pediatrics, University of Minnesota, Minneapolis, MN, United States; ^2^ Department of Pediatrics, Children’s Hospital of Philadelphia, Philadelphia, PA, United States; ^3^ Department of Pediatrics, Children’s Hospital of Colorado, Denver, CO, United States; ^4^ Department of Pediatrics, Duke University, Durham, NC, United States; ^5^ Department of Nephrology and Hypertension, Mayo Clinic, Rochester, MN, United States; ^6^ Department of Pediatrics, Emory University, Atlanta, GA, United States; ^7^ Department of Internal Medicine, University of Cincinnati, Cincinnati, OH, United States; ^8^ Department of Pediatrics, University of Iowa, Iowa, IA, United States; ^9^ Division of Nephrology and Hypertension, Cincinnati Children’s Hospital, Cincinnati, OH, United States; ^10^ Department of Pediatrics, University of Cincinnati, Cincinnati, OH, United States; ^11^ The Kidney Center at Hennepin Healthcare, Hennepin Health, Minneapolis, MN, United States; ^12^ Department of Medicine, University of Minnesota, Minneapolis, MN, United States; ^13^ Department of Surgery, University of California, Davis, Davis, CA, United States; ^14^ Department of Surgery, Icahn School of Medicine at Mount Sinai, Mount Sinai Hospital, New York, NY, United States; ^15^ Heersink School of Medicine, Department of Pediatrics, School of Medicine, University of Alabama, Birmingham, AL, United States; ^16^ Department of Pediatrics, Seattle Children’s Hospital, Seattle, WA, United States; ^17^ Department of Surgery, University of Michigan Health, Ann, Arbor, MI, United States; ^18^ Department of Internal Medicine, University of Iowa, Iowa City, IA, United States; ^19^ Clinical and Translational Science Institute, University of Minnesota, Minneapolis, MN, United States; ^20^ Department of Pediatrics, Northwestern University, Ann & Robert H. Lurie Children’s Hospital, Chicago, IL, United States

**Keywords:** focal segmental glomerulosclerosis, recurrent disease, nephrotic syndrome, kidney transplant, plasmapheresis

## Abstract

**Background:**

Focal segmental glomerulosclerosis (FSGS) is a common cause of end-stage kidney disease requiring kidney transplantation and can recur in the allograft in 30-80% of recipients resulting in reduced graft survival. Plasmapheresis has shown efficacy in treating some cases of recurrent FSGS but isolated plasmapheresis has not demonstrated efficacy in preventing recurrent FSGS. Rituximab has had anecdotal success in preventing recurrence in a single center study but has not been studied in combination with plasmapheresis for preventing FSGS recurrence.

**Methods:**

We are conducting a randomized, controlled, multicenter clinical trial of adult and pediatric kidney transplant recipients with primary FSGS to assess whether plasmapheresis in combination with rituximab prevents recurrent disease post-transplantation.

**Discussion:**

Rituximab combined with plasmapheresis is a promising, novel therapy to prevent recurrent FSGS, a disease with limited therapeutic options and no consensus guidelines for prevention or treatment.

**Clinical trial registration:**

https://clinicaltrials.gov/ct2/show/NCT03763643, identifier NCT03763643.

## Introduction

Focal segmental glomerulosclerosis (FSGS) is a clinicopathologic entity that is characterized by proteinuria +/- nephrotic syndrome, focal scarring and segmental sclerosis of the kidney glomeruli on light microscopy, fusion and effacement of the podocyte foot processes and progressive chronic kidney disease. Initial biopsies of children with nephrotic syndrome may show MCD initially followed by FSGS pathology as the disease progresses. Landmark studies over the past 15 years have identified more than 50 forms of monogenic FSGS. However, over 70% of children and approximately 80% of adults do not have an identifiable genetic cause. Non-genetic, primary FSGS is most likely caused by a circulating permeability factor as evidenced by several observations (1). At this time, the identity of the permeability factor remains unknown. There is currently no effective treatment for FSGS and the majority of patients will progress to end stage kidney disease (ESKD) requiring dialysis or kidney transplantation. Unfortunately, those patients without a monogenic cause of disease are at high risk for recurrence of FSGS after kidney transplant.

The rate of recurrence following kidney transplantation in patients with FSGS ranges between 30-50% in most studies, and in a subset of patients it may be as high as 80% (2, 3). True epidemiological estimates are not clear since many studies have included patients without genetic testing or were performed prior to the widespread availability of genetic testing. Recurrence of FSGS can occur rapidly, within minutes of transplantation, and can lead to immediate onset of proteinuria and graft dysfunction. Recurrent FSGS is definitively diagnosed with a kidney biopsy. Early kidney biopsies in recurrent FSGS often demonstrate isolated extensive foot process effacement with the classic focal sclerosis appearing only after months of proteinuria. Recurrent FSGS is the single most important cause of graft failure in patients with FSGS. Patients with recurrent FSGS have significantly decreased graft survival compared to patients without recurrence and often undergo multiple kidney transplants in their lifetime (4). They may also be declined for repeated kidney transplantation if prior allografts have been lost to recurrent FSGS.

A number of clinical risk factors for recurrent FSGS have been identified including prior recurrence, European ancestry, rapid progression of primary FSGS to ESKD within 3 years, mesangial hypercellularity on primary biopsy and living related donor transplant; however these findings are inconsistent among various small studies (2, 5). A recent study from Europe identified late steroid-resistant FSGS (nephrotic syndrome that becomes resistant to steroids after an initial therapeutic response) as a strong risk factor for recurrence (3). These findings have been replicated in our recent retrospective study of a North American cohort from the Pediatric Nephrology Research Consortium (N=116) that showed that the risk of recurrent FSGS was 71% in children with late steroid-resistant FSGS compared to 36% in children with initial steroid-resistant FSGS (6). Genetic risk factors for late steroid-resistant FSGS are unknown. In the same study, we identified native kidney biopsy findings that may predict risk of recurrence. Unfortunately, an individual’s risk of recurrent disease cannot be predicted accurately prior to transplant. Thus, patients with FSGS have a high risk of recurrent disease post-transplant that is difficult to predict and leads to poor outcomes.

Post-recurrence treatment with plasmapheresis or rituximab, although often utilized, is not effective in all patients. Effective therapies and prevention strategies for recurrent FSGS are lacking (7–10). The demonstration of a possible causative circulating factor by Savin et al. (11) galvanized the field to attempt plasmapheresis to remove the permeability factor as a therapy for recurrent FSGS. Thereafter, recurrent FSGS has most often been treated with plasmapheresis to remove the as yet undetermined circulating factor, which is effective in a subset of patients. The results of uncontrolled single center case reports and series suggest a benefit from plasmapheresis (12–14), although graft loss from recurrent disease remains unacceptably high. Others who do not respond to plasmapheresis have been treated with alternate agents such as rituximab, abatacept, or high dose cyclosporine with variable responses and are at risk for rapid progression to graft failure (15–17). Ideally, we need to identify strategies to prevent recurrent FSGS before it occurs, which will lead to longer kidney transplant survival and reduced health care costs.

No treatments are currently available to prevent the recurrence of FSGS in kidney transplant. Since 2005, there have been 3 published studies that have utilized a variety of plasmapheresis protocols for the prevention of recurrent FSGS post-kidney transplant in children with inconsistent results (18–20). The University of Minnesota pediatric transplant program has utilized pre-emptive plasmapheresis in children at risk for recurrent FSGS since 2006. In a recent review of our data, the incidence of recurrent FSGS post-transplant was not significantly different in patients who had undergone pre-emptive prophylactic plasmapheresis versus historic controls who had not (31% vs. 23%, p 0.5) (21). Although retrospective, the data suggest that plasmapheresis alone may be insufficient to prevent FSGS recurrence. Thus, other preemptive strategies to prevent recurrent FSGS are needed.

Rituximab has been used to treat recurrent FSGS and is a promising novel therapy to prevent recurrent disease. Rituximab is a chimeric monoclonal antibody against CD20 on B cells that leads to B cell depletion and has been used successfully for the treatment of primary steroid-sensitive nephrotic syndrome and steroid-resistant FSGS (22, 23). Rituximab may act in FSGS via alterations in B-cell/T-cell interactions leading to changes in cytokine secretion or co-stimulation. Alternately, rituximab may directly affect podocyte structure and function as it has been found to bind directly to the sphingomyelin phosphodiesterase acid-like 3b protein on the surface of podocytes (24). Recent systematic reviews on the use of rituximab in the treatment of post-transplant FSGS (either with or without plasmapheresis) demonstrate partial or complete remission in ~60% of treated patients (15, 25). The use of preemptive rituximab has been reported to be effective in preventing recurrent FSGS in 4 patients at very high risk due to prior graft loss from recurrent disease; however randomized controlled trials have not been performed (26). There is biological plausibility for the efficacy of preemptive treatment with rituximab, an antibody depleting therapy. Several potential permeability factors proposed as pathogenic in recurrent FSGS are antibodies directed against glomerular antigens (27). Recently, autoantibodies to the podocyte proteins nephrin and annexin A2 have been identified in patients with primary nephrotic syndrome, including patients with FSGS (28, 29). Given the effectiveness of rituximab in the treatment of some patients with both primary and recurrent FSGS and the report of successful preemptive use of Rituximab in a small series of high-risk transplant patients, we propose that treatment with Rituximab combined with plasmapheresis in patients with FSGS prior to kidney transplant is a promising novel therapy to prevent recurrent disease.

## Methods and analysis

### Study design

**Figure 1:**
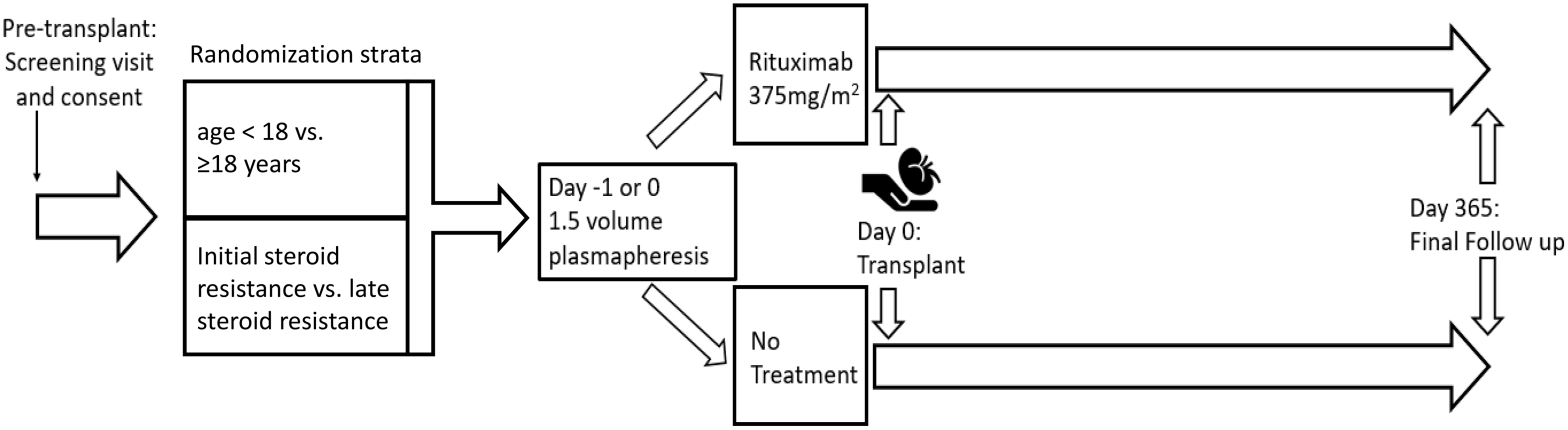
Schematic of randomized trial of Rituximab and plasmapheresis to prevent recurrent FSGS.

This is an investigator initiated, multicenter, randomized, open-label, clinical trial. A schematic outline of the study is provided in **Figure 1**. Participants will be recruited from among 14 participating pediatric and/or adult kidney transplant centers in the United States.

### Study population

This study will enroll 160 children and adults age 1-65 years at the time of transplant with biopsy-proven FSGS or minimal change disease (MCD) in their native kidney biopsy. Participants with either planned living or deceased donor undergoing their first, second or third kidney transplant will be included. Patients will be excluded if they have known monogenic FSGS or a secondary cause of FSGS (parvovirus infection, HIV, etc). They will also be excluded if they have received rituximab within 1 year prior to transplant.

### Randomization and study treatment

All participants will receive one 1.5 volume plasmapheresis session with fresh frozen plasma replacement prior to kidney transplant on day 0 or -1 as is able to be scheduled. In addition, participants will be randomized 1:1 to receive rituximab 375 mg/m^2^ intravenously following plasmapheresis with appropriate premedication of acetaminophen and diphenhydramine or to no additional therapy (US FDA IND #137324). Randomization will be stratified by history of initial steroid resistance vs. late steroid-resistant nephrotic syndrome given the significantly different risks of relapse in these two groups as well as by age (age < 18 vs. ≥18 years) (3). The randomization process will be centrally administered. DNA sample will be banked for future genetic analysis.

### Kidney transplantation

Kidney transplantation will be performed per local center protocols for induction and maintenance immunosuppression and after care. Induction immunosuppression (e.g. thymoglobulin) will be given after plasmapheresis and rituximab.

### Follow up monitoring

Participants will be followed clinically for 1 year after the date of kidney transplant. See **Table 1** for study procedures. Urine protein to creatinine ratio, serum creatinine, height, and blood pressure will be recorded at least weekly during the initial month post-transplant, monthly until 3 months post-transplant, and then every 3 months until the end of study. It is recognized that many centers will monitor urine protein more often than this schedule and these standard of care values will also be recorded. Kidney biopsies will be performed as clinically indicated and as recurrent disease is documented. CD19 count will be monitored locally at 1 month, 6 months, and 1 year post-transplant to monitor effectiveness of B-cell depletion. Recurrent FSGS will be treated per local protocols.

**Table 1:** Schedule of procedures.

Procedures	Screening/Enrollment Visit Day -2 to -365 (in-person)	Infusion Visit Visit 1, Day -1 or 0 (in-person)	Transplant Day, Study Visit 2 Day 0 (chart review)	Study Visit 3 Day 7+/-3 day (chart review/phone)	Study Visit 4 Day 14+/-3 day (chart review/phone)	Study Visit 5 Day 21 +/-3 day (chart review/phone)	Study Visit 6 Day 28+/-3 day (in-person)	Study Visit 7 Day 60 +/-7 day (chart review/phone)	Study Visit 8 Day 90 +/-7 day (chart review/phone)	Study Visit 9 Day 180 +/-14 day (in person)	Study Visit 10 Day 270 +/-14 day (chart review/phone)	Final Study Visit 11 Day 365 +/-14 days (in-person)
Informed consent/assent	X											
Demographics	X											
Medical history, initial and update	X	X	X	X	X	X	X	X	X	X	X	X
Randomization		X										
Administer study intervention		X										
Concomitant medication review	X	X	X	X	X	X	X	X	X	X	X	X
Physical exam	X	X										
Vital signs, weight	X	X					X			X		X
Height	X	X	X	X	X	X	X	X	X	X	X	X
Clinic BP^1^	X	X					X			X		X
Adverse event review and evaluation		X	X	X	X	X	X	X	X	X	X	X
Complete Case Report Forms (CRFs)	X	X	X	X	X	X	X	X	X	X	X	X
Laboratory												
Pregnancy test (SOC)		X^2^										
HepBSAg, antiHBc (SOC)	X^3^											
Serum chemistry (creatinine, albumin) (SOC)				X	X	X	X	X	X	X	X	X
Urine protein to creatinine ratio (mg/mg) (SOC)		X		X	X	X	X	X	X	X	X	X
CBC (SOC)		X		X	X	X	X	X	X	X	X	X
CD19 (absolute and %)							X			X		X
IgG, IgM, IgA		X					X			X		X
DNA sample	X											

### Safety monitoring

Rituximab has a well described safety profile. Approximately one third of treated children have acute infusion reactions consisting of fever, nausea/vomiting, diarrhea, rash, and bronchospasm. Premedication will lessen this risk. If infusion reactions occur, they generally respond to interruption of the infusion and resumption at a lower rate. Additional risks of rituximab include cytopenias and this will be monitored with CBC and differential counts which are routinely monitored post-transplant. Hypogammaglobulinemia is a known risk of rituximab and will be monitored at baseline, 3 months, 6 months, and 12 months. Potentially serious and life-threatening side effects include infections, pulmonary fibrosis, Pneumocystis jiroveci pneumonia, malignancy, and progressive multifocal leukoencephalopathy. Adverse events and serious adverse events will be recorded.

### Data safety monitoring board

Study oversight will be the responsibility of the PIs and a Data and Safety Monitoring Board (DSMB) composed of members with expertise in pediatric nephrology, adult nephrology, and statistics. The DSMB will meet at least every 6 months to assess safety and efficacy data, study progress, and data integrity for the study.

### Definition of recurrent FSGS

Recurrent FSGS will be defined as kidney biopsy with >50% foot process effacement or a urine protein to creatinine ratio >2 g/g for 2 or more consecutive days and rising with serum albumin < 2.5g/dL for those clinically unable to have a kidney biopsy. Date of recurrent FSGS will be defined as date of biopsy, date meeting clinical criteria above, or date of initiation of therapy for recurrent FSGS, whichever comes first.

### Primary outcome

Recurrent FSGS at one year post-transplant.

### Secondary outcomes

1. Time to FSGS recurrence
2. Graft survival at one year post-transplant
3. Patient survival at one year post-transplant
4. Graft function (eGFR) at one year post-transplant
5. Acute rejection at one year post-transplant
6. Proportion with CD19^+^ <1% at 1 month, 6 months, and 12 months

### Sample size

Our recent retrospective study from the Pediatric Nephrology Research Consortium demonstrated an overall recurrence rate of 43% in children with FSGS, including patients both with and without monogenic cause of disease (35.8% in patients with initial steroid-resistant FSGS vs. 70.6% in patients with late steroid-resistant FSGS) (6). Sample size estimates are based on 80% power to detect the described difference as statistically significant at a significance level of 0.05. Seventy subjects per group (140 in total) are required to detect as significant a decrease in the rate of recurrent disease from 43% in the control group to 21% in the treatment group (50% reduction). We will target enrollment of 160 patients to account for ~15% attrition or loss to follow up.

### Statistical plan and data analysis

Demographic and baseline characteristics of study participants will be summarized using descriptive statistics and compared between treatment groups using two-sample t test for continuous variables and Chi-square test for categorical variables.

The proportion of patients with recurrent disease at 1-year post-transplant (the primary outcome) will be compared between treatment groups using Chi-square test. Multivariate logistic regression model will be constructed to model the probability of recurrence. The model will include treatment group, center, and covariates determined by the investigators to impact recurrent and/or those that are significantly different between groups at baseline. Binary secondary outcomes (graft survival, patient survival, acute rejection, and CD19^+^ <1%) will be analyzed similarly. Survival analysis will be performed to evaluate the effect of treatment on time to FSGS recurrence. Estimated GFR, a continuous secondary outcome, will be compared between groups using two-sample t test. Multivariate linear regression analysis will be conducted to examine the effect of treatment on eGFR adjusting for covariates.

Intention-to-treat analysis will be used. Analysis will be performed using Statistical Analysis Software (version 9.3, SAS Institute Inc., Cary, NC). A two-sided p-value < 0.05 will denote statistical significance.

## Discussion

It is not uncommon for an initial native biopsy on a patient with steroid resistant nephrotic syndrome to show MCD, only to have subsequent biopsies for disease progression demonstrate FSGS. One series of 77 transplants for FSGS showed that 13% had MCD on an initial native biopsy with FSGS on a subsequent biopsy (30). Recurrent disease was equally likely to occur with MCD or FSGS on initial biopsy, therefore we have included both in this trial.

One limitation of this trial is that genetic testing is not required to exclude those with genetic causes of nephrotic syndrome that would be unlikely to recur. Pediatric centers routinely perform genetic testing for all patients with FSGS and those with genetic cause would be excluded for this trial, however this is not the case for all adult transplant centers and is currently not recommended by KDIGO guidelines. Similarly, enrollment in this study requires the judgement of the clinical investigator to exclude patients with typical secondary causes of FSGS. The inclusion and exclusion criteria are consistent with the field’s current standard for evaluation of FSGS and aim to enrich the study population with those who are more likely to have recurrent disease. An additional potential limitation of this study is that the power calculation was performed using data from a pediatric population given lack of consistent data in the adult FSGS population during the study design phase. Since study initiation, a large multicenter review described a recurrence rate in adults with primary FSGS to be 37% in a US population, slightly lower than 42% recurrence rate in pediatric patients which may lead to an underpowered study (31).

Several participating centers were offering preemptive plasmapheresis to patients at high risk of recurrent FSGS prior to the initiation of this trial. We chose to add Rituximab to this baseline given the complexity of requiring a center to remove a therapy they were already offering. This trial was designed to include a single episode of pheresis with a single dose of rituximab prior to transplant as a pragmatic decision to allow for therapy to be completed during the short window prior to deceased donor kidney transplantation. Future studies would be required to evaluate whether there is a potential benefit from additional pre-transplant doses of rituximab and to determine the optimal number of plasmapheresis sessions, if any.

The purpose of this study is to test the effectiveness of rituximab in combination with plasmapheresis prior to kidney transplant, in reducing the incidence of recurrent FSGS after kidney transplantation. In recipients in whom FSGS does recur, we will study the impact of these interventions on the time to FSGS recurrence. In addition, we will be monitoring transplant outcomes (graft survival rate, rejection rate and estimated GFR at 1 year post-transplant) and patient outcomes (patient survival at one year post-transplant). The safety of plasmapheresis and rituximab will be assessed. Since studies in nephrotic syndrome have demonstrated that the rituximab effect is lost with recovery of the CD19+ cell population (32), we will study the proportion of patients with CD19^+^ <1% at 1 month, 6 months, and 12 months in the rituximab arm of the study. Single center studies have had conflicting results on the effectiveness of preemptive plasmapheresis on rate of recurrence of FSGS; however a recent meta-analysis did not demonstrate benefit of plasmapheresis alone (33). Similarly, single-center primarily retrospective studies of rituximab for prevention of recurrent FSGS have had mixed results with some suggesting benefit (24, 26, 33). We have designed a first of its kind randomized controlled multicenter study in adult and pediatric kidney transplant candidates with primary FSGS as the cause of end-stage kidney disease.

### Study progress to date

The success of this study depends on recruitment and adherence to the randomization protocol. Recruitment in previous clinical trials of FSGS was impeded by stringent definitions of primary FSGS, an overestimation of anticipated case number at each site, and lack of site investigator enthusiasm for the study intervention (34, 35). To mitigate these issues, we allowed site investigators to use their discretion to determine if a patient had primary or secondary FSGS and utilized SRTR 3-year data on ESKD etiology for case number prediction at each site.

Due to the COVID-19 pandemic, most transplant centers in our study had inactivated their transplant programs for varying periods of time. Responsively, we halted recruitment in March 2020 and reopened in June 2020, although the extent and timing of study closure varied by site. One of our centers subsequently withdrew from participation due to lack of research personnel for non-COVID-19 study purposes. One of our study sites withdrew from the study due to center rules which precluded research coordinators from coming to the hospital for non-COVID-19 studies.

Given the delays in study recruitment due to the COVID-19 restrictions, a number of changes to the initial inclusion criteria were made to allow for more rapid recruitment. The initial inclusion criteria were restricted to children and adults age ≤ 40 years to avoid the inadvertent inclusion of secondary FSGS which would confound results. Our study site investigators in adult programs were concerned that eligible patients would be eliminated with such stringent inclusion age criteria and so these were expanded to include those under 65 years of age starting in July 2021. Similarly, the initial protocol allowed inclusion of transplant recipients at the time of their first or second transplant. After feedback from sites and coordinators, this criteria was expanded to include patients receiving their third transplant as well in July 2021.

## Ethics statement

The studies involving human participants were reviewed and approved by University of Minnesota, Children’s Hospital of Philadelphia, Children’s Hospital of Colorado, Duke University, Mayo Clinic, Emory University, University of Cincinnati, University of Iowa, Cincinnati Children’s Hospital, Hennepin Health, University of California-Davis, University of Alabama at Birmingham, Seattle Children’s Hospital, University of Michigan, Lurie Children’s Hospital. Written informed consent to participate in this study was provided by the participants’ legal guardian/next of kin. Written informed consent and/or assent to participate in this study was provided by the participant or the participants’ parent/legal guardian.

## Author contributions

MR & PV drafted the initial manuscript. MR, BC, PV, RaG, and QW obtained grant funding from the Department of Defense. All remaining authors are site PIs and read, revised, and approved the final manuscript. All authors contributed to the article and approved the submitted version.
